# Protection mediated by chemokine CXCL10 in BALB/c mice infected by *Leishmania infantum*

**DOI:** 10.1590/0074-02760160529

**Published:** 2017-08

**Authors:** Webertty Mayk Eufrásio Figueiredo, Sayonara de Melo Viana, Dorotheia Teixeira Alves, Priscila Valera Guerra, Zirlane Castelo Branco Coêlho, Helene Santos Barbosa, Maria Jania Teixeira

**Affiliations:** 1 Universidade Federal do Ceará, Faculdade de Medicina, Departamento de Patologia e Medicina Legal, Fortaleza, CE, Brasil; 2 Universidade Federal do Ceará, Faculdade de Farmácia, Odontologia e Enfermagem, Departamento de Análise Clínica, Fortaleza, CE, Brasil; 3 Fundação Oswaldo Cruz-Fiocruz, Instituto Oswaldo Cruz, Laboratório de Biologia Estrutural, Rio de Janeiro, RJ, Brasil

**Keywords:** L. infantum, CXCL10, mice, nitric oxide, cytokines, macrophages

## Abstract

**BACKGROUND:**

Visceral leishmaniasis (VL) caused by *Leishmania infantum* is characterised by the loss of the ability of the host to generate an effective immune response. Chemokines have a direct involvement in the pathogenesis of leishmaniasis, causing a rapid change in the expression of these molecules during infection by *Leishmania*.

**OBJECTIVES:**

Herein, it was investigated the role of CXCL10 in controlling infection by *L. infantum*.

**METHODS:**

RAW 264.7 macrophages were infected with *L. infantum in vitro* and treated or not with CXCL10 (25, 50 and 100 ng/mL). Parasite load, as well as nitric oxide (NO), IL-4 and IL-10 production were assessed at 24 and 48 h after infection. *In vivo*, BALB/c mice were infected and treated or not with CXCL10 (5 μg/kg) at one, three and seven days of infection. Parasite load, IFN-g, IL-4, TGF-β and IL-10 were evaluated one, seven and 23 days post treatment.

**FINDINGS:**

*In vitro*, CXCL10 reduced parasitic load, not dependent on NO, and inhibited IL-10 and IL-4 secretion. *In vivo*, CXCL10 was able to reduce the parasite load in both liver and spleen, four weeks after infection, representing a higher decrease in the number of parasites in these organs, also induced IFN-γ at day 23 after treatment, correlating with the decrease in parasite load, and reduced IL-10 and TGF-β.

**MAIN CONCLUSIONS:**

This study suggests a partial protective role of CXCL10 against *L. infantum*, mediated by IFN-g, not dependent on NO, and with suppression of IL-10 and TGF-β. These data may provide information for the development of new approaches for future therapeutic interventions for VL.

Visceral leishmaniasis (VL) is caused by protozoa of the genus *Leishmania*, in the Americas by *L. chagasi* (*L. infantum* synonymy) (Maurício et al. 2000) and in the Old World by *L. donovani* and *L. infantum* (Piscopo & Mallia 2006). These species, known as viscerotropic, preferably infect macrophages and dendritic cells in the bowels, and parasites are promptly found in the liver, spleen and bone marrow (Wilson et al. 2005). The clinical manifestations of the disease can vary from asymptomatic infection to a progressive visceral disease, which if untreated can lead to death and is characterised by fever, weight loss, hypergammaglobulinemia, hepatosplenomegaly, anemia, thrombocytopenia, leukopenia and immunosuppression (Piscopo & Mallia 2006).

Several experimental models of VL in rodents have been developed, and although none completely reproduced the human disease, the studies using these models have been shown to be very important because they allow investigating the immunological mechanisms and histopathological aspects of the disease (Wilson et al. 2005, Nieto et al. 2011). BALB/c mice, when infected by *L. donovani* or *L. infantum*, can resolve the infection spontaneously after some time. In these animals, the effect of IL-12 is delayed for four weeks after infection, at which occurs the appearance of lymphocytes producing IFN-γ antigen-specific, granuloma formation in the liver, and development of an antigen-specific response of CD4+ and CD8+ cells (Wilson et al. 2005). A remarkable feature of the experimental infection of mice with the viscerotropic species of *Leishmania* is the distinct organ-specific immune response. An acute infection occurs in the liver, which can be resolved between the 4th and 6th week of infection, with subsequent immunity to re-infection, while in the spleen, the parasites may persist (Stanley & Engwerda 2007). The control of liver disease in mice requires a coordinated response of the host, involving the development of granulomas around the infected macrophages (Murray et al. 2002, Nieto et al. 2011). In contrast, spleen and bone marrow become chronically infected by mechanisms that are not well understood. In the spleen, the persistence of the parasite is accompanied by a failure in the formation of granulomas, splenomegaly, and disruption of lymphoid tissue microarchitecture (Wilson et al. 2005, Stanley & Engwerda 2007, Nieto et al. 2011).

Several studies have shown that chemokines have a direct involvement in the pathogenesis of leishmaniasis, causing a rapid change in the expression of these molecules during infection by *Leishmania* (Teixeira et al. 2006). In the liver, at the beginning of infection, parasitised Kupffer cells secrete chemokines such as CCL3, CCL2 and CXCL10, which stimulate the recruitment of monocytes and granulocytes (Stanley & Engwerda 2007). CXCL10 belongs to a large subpopulation of cytokines, which are critical mediators to the function of leukocytes, polarisation of Th1 cells, and activation and trafficking of cells involved in inflammatory responses (Teixeira et al. 2006). CXCL10 binds with high affinity to its CXCR3 receptor (Chen et al. 2004), known to be expressed in many cell types, including CD4+, CD8+, memory cells, NK cells and some subpopulations of dendritic cells (Mohan et al. 2005). Injection of CXCL10 in BALB/c mice infected with *L. major* has been shown to induce a strong recruitment and activation of NK cells (Müller et al. 2001). Exogenous CXCL10 is able to reduce the parasitic load of macrophages infected with *L. amazonensis in vitro*, and in BALB/c mice infected with *L. amazonensis*, can decrease the size of the lesion and parasite load, followed by an increase in IFN-g, IL-12 and nitric oxide (NO) (Vásquez & Soong 2006). In VL, studies in BALB/c mice have shown that CXCL10 is involved in the protective response of infection by *L. donovani*, inducing a Th1 response through the regulation of the pathway of inflammatory mediators, such as NO and inflammatory cytokines (Gupta et al. 2009). CXCL10 may participate in the protection against *L. donovani* with remarkable decrease of immunoregulatory cytokines, IL-10 and TGF-b, secreted by T CD4+cells (Gupta et al. 2011).

Herein, it was assessed whether treatment with CXCL10 would confer protection against infection by *L. infantum* in macrophages and in BALB/c mice.

## MATERIALS AND METHODS

*Animals* - A total of 108 BALB/c mice, male, eight weeks old, obtained from the Biotério Central do Departamento de Patologia e Medicina Legal da Universidade Federal do Ceará, CE, Brazil (DPML/UFC-CE), were used in the experiments. The animals were maintained at 25ºC with appropriate commercial ration and water *ad libitum*. The project was approved by Comitê de Ética em Pesquisa Animal (CEPA) at the UFC, under registration number 52/2014.

*Parasites* - Promastigotes of *L. infantum* (MHOM/BR/BA-262) were maintained in a golden hamster. After being recovered from the animal, the parasites were cultured at 25ºC in N.N.N. medium, containing Schneider’s insect medium (Sigma-Aldrich) supplemented with 20% fetal bovine serum (Sigma-Aldrich), 2% sterile human urine and antibiotics (100 U/mL penicillin and 100 mg/mL streptomycin) (all Sigma-Aldrich). The virulence of the strain was maintained by regular passage in a golden hamster. For infection, the parasites were used until the 5th passage, *in vitro*.

*In vitro assays with macrophages* - RAW 264.7 macrophages culture were centrifuged at 400 x *g* for 15 min at 5ºC, and the cell suspension was adjusted in RPMI 1640 medium supplemented with 10 mM HEPES, 100 U/mL penicillin, 100 µg/mL streptomycin, and 2 mM L-glutamine (all from Sigma-Aldrich). Then, the cells were distributed in 24-well plates, each well containing a round glass coverslip of 23 mm at a concentration of 1x10^6^ cells/coverslip. Macrophages were incubated for 24 h with 5% CO_2_ at 37ºC and 95% humidity. Cells not adhered were removed by washing with warm RPMI (37ºC; three times) and cultured with RPMI supplemented with or without *L. infantum* live promastigotes, a ratio of 10:1 cell parasites for 12 h. Extracellular parasites were removed by washing (three times) with warm RPMI (37ºC). After this period, the cells were treated with 16 mg/mL of pentavalent antimony (Glucantime), CXCL10 (25, 50 and 100 ng/mL) and LPS (20 ng/mL; positive control) and then were cultured for 24 and 48 h at 37ºC, 5% CO_2_ and 95% humidity. Culture supernatants were collected at 24 and 48 h to the dosage of cytokines and NO.

*Determination of macrophage infection* - To quantify the level of infection of macrophages, coverslips containing the cells were washed with saline, and then fixed and stained with Giemsa (Sigma-Aldrich). Stained coverslips were mounted on slides and examined under optical microscopy at a magnification of 100 X. The slides were examined to determine the number of amastigotes/50 macrophages.

*NO assay* - Macrophages culture supernatants were tested for NO release in the form of nitrite (NO_2_) by the Griess reaction. The supernatants were incubated with freshly prepared Griess reagent (1:1 v/v) for 10 min at room temperature. The absorbance was measured spectrophotometrically at 540 nm and the NO_2_ concentration determined using a standard curve of sodium nitrite and expressed as µmol/mL. To avoid interference from NO_2_ possibly present in the medium, a white test was performed for each experiment.

*Infection and treatment with CXCL10 in BALB/c mice* - Metacyclic promastigotes at the stationary phase were inoculated intraperitoneally into BALB/c mice (n = 36) at a concentration of 2 x 10^7^ parasites in 20 µL of saline. The animals were divided into two groups, with 18 animals each. One group received CXCL10 (5 μg/kg) (Thermo Scientific) and the other received saline (Untreated group) intraperitoneally after 1, 3 and 7 days of infection (Gupta et al. 2009). The animals were euthanised after 1, 7 and 23 days of treatment by inhalation of Halothane (Sigma-Aldrich). Six animals per group were euthanised on each period. The animals were weighed before starting treatment and every euthanasia to achieve a weight analysis.

*Determination of parasite numbers in liver and spleen* - The number of parasites in the liver and spleen was quantified using the technique of limiting dilution as previously described (Titus et al. 1985). Briefly, the animals were euthanised and submerged in 3% iodised alcohol up to 3 min to allow decontamination. Then the spleen and liver were removed aseptically, weighed, and one fragment from each organ (spleen: from 0.067 to 0.070 g; liver: from 0.26 to 0.30 g) was homogenised in 1 mL of Schneider medium and left to rest for 5 min. Eight dilutions were made from this cell suspension (10, 50, 100, 500, 10^3^, 10^4^, 10^5^ and 10^6^) in Schneider’s medium supplemented with 20% FBS, 2% sterile human urine, and antibiotics (100 U/mL penicillin and 100 μg/mL streptomycin) (All from Sigma-Aldrich). One hundred microliters of these dilutions were distributed into 96-well plates, flat bottom, and six wells/dilution. The plates were sealed and incubated at 25ºC for three weeks. The wells were observed on an inverted optical microscope (Nikon) every 3 days to record the dilutions containing promastigotes. The final number of parasites was determined using the ELIDA 12c software.

*Culture of splenocytes* - Spleen cells were isolated from animals infected with *L. infantum* promastigotes, treated or untreated with CXCL10 (six animals per group) and separated by centrifugation with Ficoll, 800 x *g* for 30 min at 25ºC, after one, seven and 23 days after treatment. The cells were washed with RPMI 400 x *g* for 15 min at 5ºC and then cultured in 96-well plates (1 x 10^6^ cells/mL) in RPMI medium supplemented with 10% fetal bovine serum (FBS), 50 μM 2-mercaptoethanol, 2 mM L-glutamine and antibiotics (100 U/mL penicillin, 100 mg/mL streptomycin) with or without live promastigotes at a concentration of 1 x 10^7^/mL, at 37ºC, 5% CO_2_. As control, cells were isolated for the spleen of non-infected BALB/c mice. The supernatants were collected after 48 h and preserved at -20ºC for subsequent dosing of cytokines.

*Cytokines assay* - Levels of cytokines obtained from both the culture of macrophages (only IL-4 and IL-10) and culture of splenocytes (IFN-γ, IL-4, IL-10 and TGF-β) were determined using the ELISA technique as recommended by the kit manufacturer (BD Biosciences). The results were analysed using the Softmax PRO software (Molecular Devices).

*Statistical analysis* - *t-*Student test was applied to verify the statistical significance between the treated and the untreated group. For comparisons between multiple groups it was used the one-way ANOVA test, followed by Bonferroni post-test. The tests were performed using GraphPad Prism Software version 5.00 (GraphPad Software, San Diego, CA, USA). The results are presented as arithmetic mean and standard error of the mean. In all tests, the minimum significance was accepted when p < 0.05.

## RESULTS

**Fig. 1:**
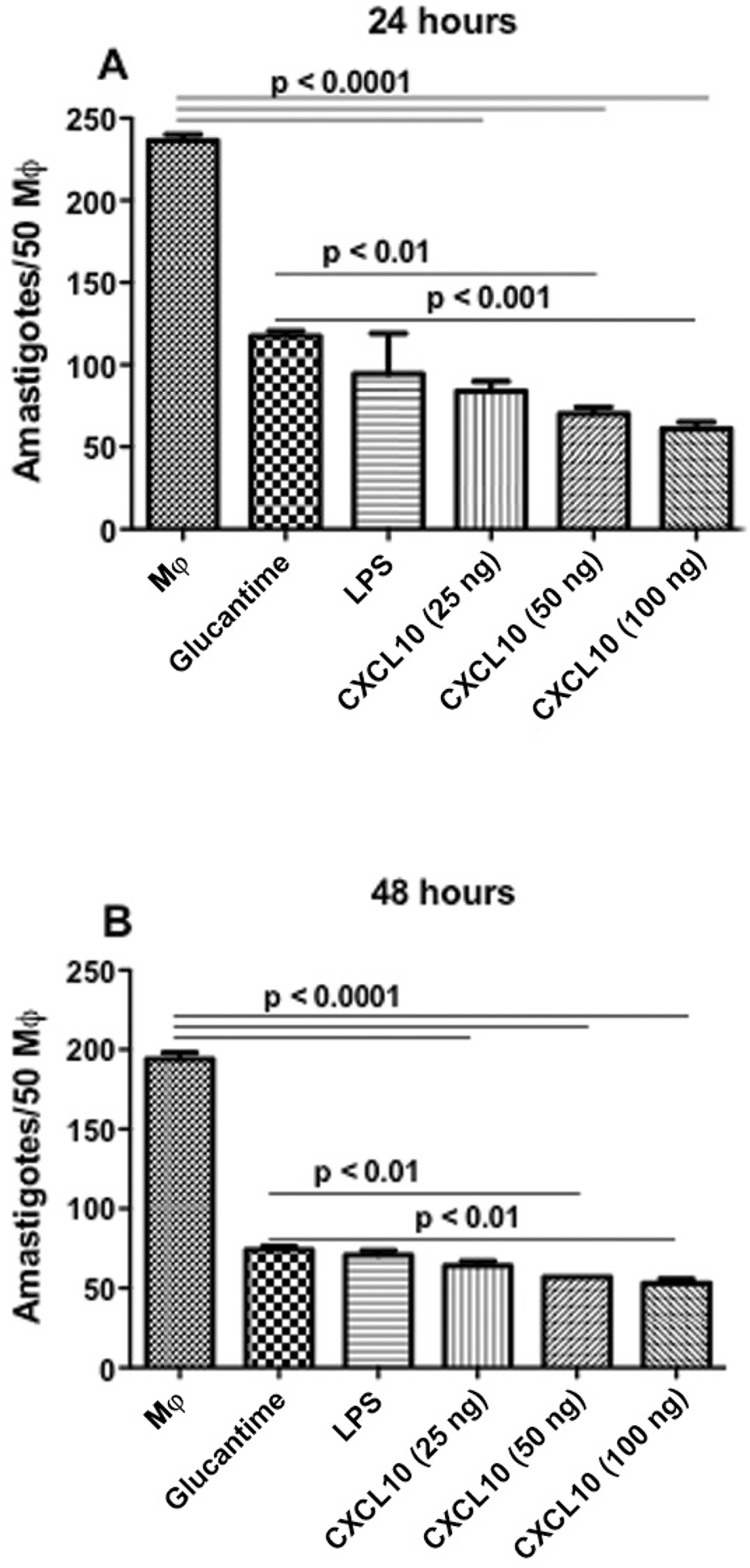
: number of parasites in macrophages infected with *Leishmania infantum* and treated or untreated with CXCL10. RAW 264.7 macrophages were infected with promastigotes of *L. infantum* (10 parasites: 1 macrophage) and after 12 h of infection the cells were treated *in vitro* with CXCL10 (25, 50 and 100 ng/mL), LPS (20 ng/mL) and Glucantime (16 mg/mL). After incubation 24 (A) and 48 h (B), the number of amastigotes per 50 macrophages was determined under an optical microscope. Results are expressed as mean ± standard error of mean, and are representative of three independent experiments.

*Parasite load in macrophages* - Treatment with CXCL10 resulted in a significant reduction of the parasitic load at concentrations of 50 ng/mL (70.3%) and 100 ng/mL (74.0%) compared to Glucantime (50.4%) and untreated animals, after 24 h (Fig. 1A). After 48 h, treatment with CXCL10 maintained reduction in the number of intracellular parasites at concentrations of 50 ng/mL (70%) and 100 ng/mL (72.6%) when compared to Glucantime (61.8%) and untreated (Fig. 1B).

**Fig. 2:**
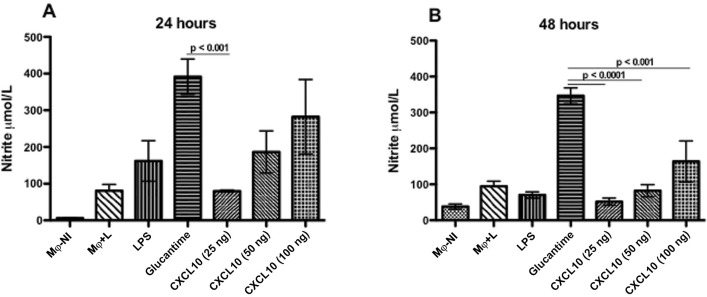
: nitric oxide (NO) by macrophages infected with *Leishmania infantum*, and treated or untreated with CXCL10. RAW 264.7 macrophages were infected and treated as described in Fig. 1. Culture supernatants were collected with 24 h (A) and 48 h (B) for the measurement of NO in the form of nitrite (NO2). The data represent the arithmetic mean ± standard error of the mean, and are representative of three independent experiments. MØ+NI = macrophage not infected; MØ+L = macrophage infected with *L. infantum.*

*NO production by macrophages* - After 24 h, NO production behaved in a dose-dependent manner in samples that were treated with CXCL10, with a production average of from 79 μmol/L to 282 μmol/L (Fig. 2A). After 48 h, there was a reduction of NO production, CXCL10 induced average production ranging from 52 μmol/L to 164 μmol/L; still showing a dose-dependence (Fig. 2B).

**Fig. 3:**
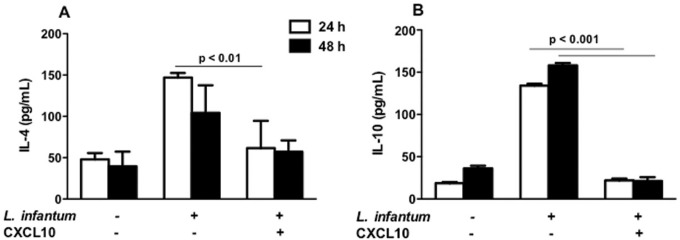
: production of IL-4 and IL-10 by macrophages infected with *Leishmania infantum* and treated or untreated with CXCL10. RAW 264.7 macrophages were infected with *L. infantum* and, after 12 h, treated *in vitro* with CXCL10 (100 ng/mL). Culture supernatants were collected with 24 h (A) and 48 h (B) for the measurement of cytokines. Results are expressed as mean ± standard error of mean, and are representative of three independent experiments.

*Cytokine production in vitro* - Treatment with CXCL10 (100 ng/mL) in macrophages infected by *L. infantum* inhibited IL-4 production (significant at 24 h post-infection; p < 0.01) and IL-10 (p < 0.001) in two time periods (Fig. 3A-B). At 48 h after infection, the results were similar for both IL-4 and IL-10 (Fig. 3A-B).

**Fig. 4:**
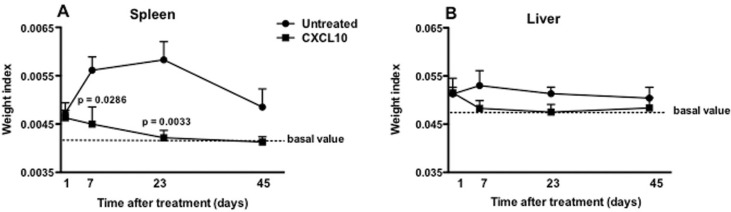
: relative weight of spleen (A) and liver (B) of BALB/c mice infected with Leishmania infantum and treated or not with CXCL10. The animals were treated with CXCL10, intraperitoneally (5 μg/kg), or saline at one, three and seven days of infection. At one, seven, 23 and 45 days after the treatment the animals were euthanised and the spleen and liver were removed. Data from six animals per group are represented by arithmetic mean ± standard error. The dashed lines represent the arithmetic mean of the relative weight of spleen (0.0042) and liver (0.048) of six healthy animals (basal value).

*Relative weight of the liver and spleen* - The analysis of the relative weight of the spleen and liver was measured one, seven, 23 and 45 days after treatment. It was observed that on the 1st day after treatment, the treated and the untreated group showed no differences in the weight of the spleen, although the weight of the spleen of both groups (untreated: 0.0056 ± 0.003; treated: 0.0045 ± 0.0004) was slightly higher than those found in healthy animals (0.0042 ± 0.0002) (Fig. 4A). There was a decrease in spleen weight of the treated animals after the 7th day, unlike what happened with the untreated group, whose weights only decreased after 45 days of treatment, and yet were above the weight of the spleen of healthy animals. When the groups were compared, it was observed that there were differences between them on 7th day (p = 0.0286) and 23 after treatment (p = 0.0033) (Fig. 4A). Regarding the liver, it was observed that the weight of this organ decreased after the 7th day of treatment in treated animals, when compared to untreated animals. Nevertheless, no difference was observed between the groups, although the liver weight in untreated animals showed a slight tendency to increase more than the treated animals, when compared with the liver weight of healthy animals (Fig. 4B).

**Fig. 5:**
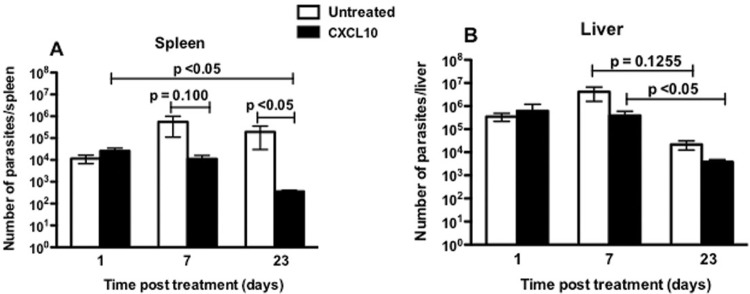
: parasite load in the spleen (A) and liver (B) of BALB/c mice infected with *Leishmania infantum* and treated or untreated with CXCL10. Animals were treated as described in Fig. 4. After one, seven and 23 days of treatment, animals were euthanised; the spleen and liver were removed and used for evaluating the parasite load. Data from six animals per group are represented by the arithmetic mean ± standard error of the mean, and are representative of three independent experiments.

*Number of parasites in the spleen and liver* - The results showed that after the 1st day of treatment, animals treated with CXCL10 showed no difference in the number of parasites in the spleen and liver when compared to untreated animals (Fig. 5A-B). As expected, the liver was the most infected organ in the early eight days of infection (means: one day after treatment), presenting in both groups (treated and untreated) a number of parasites near the 10^6^ (Fig. 5B). On the other hand, it was found in the spleen approximately half of the initial inoculum, about 10^4^ parasites in both groups (treated and untreated) (Fig. 5A). After seven days of treatment, the number of parasites in treated animals remained stable in both the spleen (approximately 10^4^) and the liver (approximately 10^6^) (Fig. 5A-B). After 23 days of treatment, there was a decline in the number of parasites in target organs in both the treated and untreated groups. However, this decrease in parasite load was found to be significant only in the spleen of the treated group (p = 0.0272), when compared to the untreated group (Fig. 5A). Even at that time, it was observed that in the treated group, in both organs, the number of parasites reached the lowest values (approximately 10^2^ in spleen and 10^3^ in the liver) (Fig. 5A-B). The group that received CXCL10 in different time periods showed that in the spleen there was a difference (p < 0.05) between 1-23 days post treatment (Fig. 5A). In the liver, there was also a significant decrease (p < 0.05) from day seven to day 23 post treatment in the treated group, showing a reduction in parasite load equivalent to that found in the spleen. Although there was an apparently similar reduction in liver rate between treated and untreated groups from day seven to day 23 post treatment, in fact, there was no statistical significance (Fig. 5B).

**Fig. 6:**
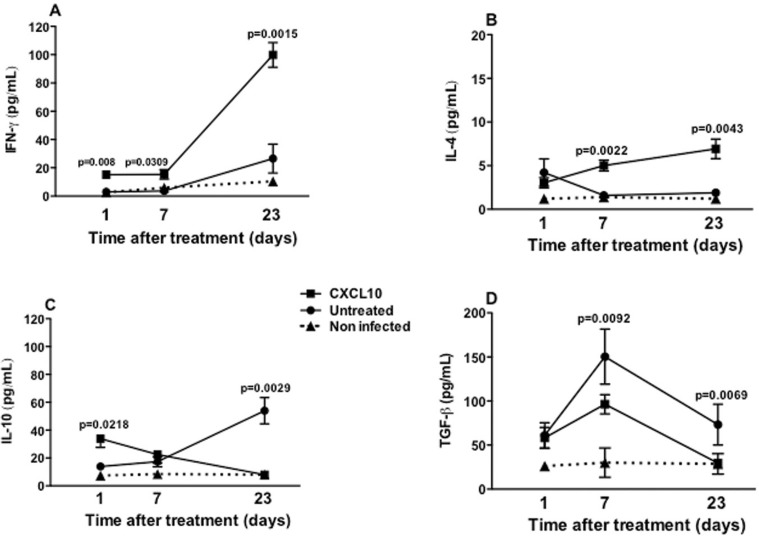
: production of IFN-γ (A), IL-4 (B), IL-10 (C) and TGF-β (D) by splenocytes from BALB/c mice infected with *Leishmania infantum* and treated (■) untreated (●) with CXCL10, or non-infected (NI) (▲). Animals were treated as described in Fig. 4. After one, seven and 23 days of treatment, the animals were euthanised to obtain the splenocytes. The supernatants of these cells were collected at 48 h for the measurement of the cytokines. Data from six animals per group are shown as the arithmetic mean ± standard error of the mean, and are representative of three independent experiments.

*Production of cytokine in vivo* - IFN-γ was produced in a more outstanding way by splenocytes from treated animals than the untreated animals, in the three time periods evaluated (after one day, p = 0.0218; seven days, p = 0.0309; and after 23 days of treatment, p = 0.0015). In animals treated with CXCL10, IFN-γ production reached its maximum value of 100 pg/mL at day 23 post treatment, which was equivalent to a five-fold increase relative to the initial concentration of 20 pg/mL (p < 0.05) (Fig. 6A). In both groups, IL-4 production remained always below of 10 pg/mL, at all three time periods evaluated (Fig. 6B). After the 1st day of treatment there was no difference between groups, however, in the last two days of the study it was observed that animals treated with CXCL10 induced a higher production of IL-4, compared with those untreated (seven days post treatment, p = 0.0022; 23 days post treatment, p = 0.0043) (Fig. 6B). In the animals treated with CXCL10, IL-10 production was twice as high as that of IFN-γ at one and 23 days after treatment, reaching a concentration of approximately 40 pg/mL, and also significantly higher than that of untreated animals (p = 0.0218) in the same time period (Fig. 6C). After seven days of treatment, there was no difference in IL-10 between CXCL10-treated and untreated animals, however, after 23 days, it fell significantly in the treated animals (p = 0.0029) (Fig. 6C). By contrast, in the untreated group, the production of IL-10 remained growing, with about 60 pg/mL being detected after 23 days of treatment (Fig. 6C). Regarding TGF-β, it was observed that the levels of this cytokine were always much higher when compared with the other cytokines evaluated. After the 1st day of treatment, both the untreated and the treated group had similar levels of TGF-β, about 50 pg/mL (Fig. 6D). After seven days of treatment, the production of TGF-β was lower in the treated group, about 100 pg/mL, lower than that produced by the untreated group (150 pg/mL; p = 0.0092) (Fig. 6D). After 23 days of treatment, the production of this cytokine decreased in both groups (treated and untreated) (Fig. 6D).

## DISCUSSION

This study evaluated the potential of CXCL10 in controlling infection caused by *L. infantum* using *in vitro* and *in vivo* models of VL. *In vitro*, CXCL10 (100 ng/mL) was able to reduce by 74.0% the intracellular parasite burden compared to infected and untreated macrophages. Also, *in vivo*, it was observed that treatment with CXCL10 was able to reduce the parasite load in both liver and spleen, four weeks after infection, representing a higher decrease in the number of parasites in these organs. This decrease in the number of parasites in the spleen correlated with the decrease in size of this organ in treated animals compared to untreated animals. During infection by *L. infantum* in mice, the amastigotes multiply rapidly during the first two weeks in the liver and disappear spontaneously around the 8th week of infection, whereas in the spleen parasites grow more slowly, leading to a chronic infection in this organ (Nieto et al. 2011). The results of parasite load found in this study corroborate the findings of the literature and more importantly, suggest a protective role of CXCL10 in infection by *L. infantum*, since this chemokine was able to induce a augmented reduction in the number of parasites in the two target organs of the treated animals, which was not observed in the untreated group.

The probable effector mechanism of this parasitic reduction is related to macrophage activation induced by CXCL10, since it has been reported that some chemokines such as CCL2 and CXCL10, can activate macrophages to participate in reducing the number of parasites (Ritter & Moll 2000). Macrophages mediate *Leishmania* destruction mainly via reactive oxygen derivatives and NO production (Vouldoukis et al. 1997). Also, it has been showed that a phenotype of *L. infantum* was resistant to death by apoptosis mediated by NO (Holzmüller et al. 2005). In this present work, the reduction of parasites *in vitro* do not seem to depend exclusively of NO, since NO production decreased significantly in infected macrophages treated with CXCL10 when compared to those infected and not treated with the chemokine. Some species of *Leishmania* are resistant to the microbicidal action of NO, as some isolates of *L. braziliensis*, which exhibited higher intracellular growth even in the presence of LPS and IFN-γ (Souza et al. 2010). There are reports that other oxygen metabolism products are involved in the respiratory burst in activated murine macrophages, such as hydrogen peroxide (H_2_O_2_) and superoxide anion (O_2_), and thus would also be involved in the elimination of *Leishmania* (Van Assche et al. 2011) which corroborates the hypothesis suggested in the present work.

In murine models of VL, using BALB/c mice, the control of the disease depends on the magnitude of the Th1 cell response, leading to the production of IFN-γ, the activation of macrophages (Kaye et al. 2004), the formation of mature hepatic granulomas as well as the production of reactive nitrogen species and oxygen intermediates, which are essential for the elimination of parasites within the Kupffer cells and dendritic cells (Murray 2001, Stanley & Engwerda 2007). The onset of the disease is associated with a decrease in the response to these factors, which helps the parasite to live and reproduce in the macrophage phagolysosome (Melby et al. 2001). It was found that IFN-γ production in the group treated with CXCL10 was augmented when compared to the untreated group in the spleen. This production was significantly increased five times than the concentration produced after the first day of treatment, an increase that coincided with the decrease in parasite load after 23 days post treatment, strengthening the hypothesis of the protective role of CXCL10. Previous studies have demonstrated the importance of IFN-γ in the control of visceral infection. Monocytes of healthy individuals previously treated with IFN-γ produce, after infection with *L. donovani*, a greater amount of TNF-α, important cytokine in the host response to microbial infection (Reiner et al. 1990). Corroborating the high levels of IFN-γ and the decrease of the parasite load, it was observed that CXCL10 induced a significant reduction in the production of IL-10 and TGF-β, regulatory cytokines, suggesting that CXCL10 induced a important Th1 response in BALB/c mice infected with *L. infantum*.

The treatment with CXCL10 *in vitro* induced a significant decrease of IL-10, anti-inflammatory cytokine and present in high levels during the active stage of the disease. This date confirms the protective response represented by the reduction of the parasitic load, even with decreased production of NO. On the animals receiving CXCL10, the concentration of IL-10 was 10 times lower than the concentration of IFN-g, however, despite CXCL10 have induced the reduction of TGF-β concentration to close proximity to the concentration of IFN-g, the cytokine was still able to promote Th1 polarisation and reduction of parasite load. These data are consistent with other studies showing that IL-10 plays an important role in suppressing the protective immune response in murine VL (Murray et al. 2003). The association of immunosuppression induced by IL-10 in human VL is well established. Patients with active disease present high serum levels of IL-10, as well as increased expression of IL-10 mRNA in lesions. IL-10 is a regulatory cytokine that is induced as a homeostatic response, which protects tissues from collateral damage caused by excessive inflammation (Mege et al. 2006).

Recently, studies have also shown that treatment with CXCL10 results in protection *in vivo* against *L. donovani*, through significant reduction of the immunosuppressive cytokines, IL-10 and TGF-β, due to modulation of regulatory T cells (Gupta et al. 2011). In the present study, the levels of TGF-β in the animals treated with CXCL10 were always much higher than those of other evaluated cytokines. These results does not fully corroborated other studies suggesting that the role of TGF-β in the control of parasite load and the resistance of the host, dependent of IFN-g, during infection by viscerotropic strains such as *L. donovani*, seems to be less important compared to IL-10 (Murray et al. 2005). Studies with animals deficient in IL-10 and TGF-β, infected with *L. infantum* and treated with CXCL10 could perhaps clarify this issue.

Unlike the models of murine cutaneous leishmaniasis, in which IL-4 is associated with the Th2 response, in the model of infection by *L. donovani* there are studies showing that IL-4 does not exacerbate the disease and may in fact act in combating the parasite, which is rather paradoxical (Mohrs et al. 1999). These studies showed that IL-4 could act positively regulating immunity against *L. donovani* by increasing the number of mature granulomas and consequent reduction of parasite load in the liver (Stäger et al. 2003). Herein, the concentration of IL-4 in both groups always remained very low at all three time periods analysed. However, in treated animals it was observed greater production of IL-4 compared with the untreated group, concomitant to the earlier presence of mature granulomas in the livers of animals that received the chemokine (data not shown).

More recently, Murray et al. (2016), in experiment performed in the *L. donovani*-infected livers of gene-deficient C57BL/6 mice (CXCL10-/- and CXCR3-/) showed that CXCL10 may promote anticipated granuloma assembly, however, may hinder early parasite control and may has no role in conversion of developing granulomas to histologically mature-appearing structures. Therefore, CXCL10 appears to paradoxically promote liver infection too.

Chemokines may act as promising new therapeutic agents to combat *L. donovani* because, among the types of cell-mediated response, some may induce polarisation of cell response of the Th1 type, able to fight infection (Gupta et al. 2009). Collectively, data from this study suggest an important protective role of CXCL10 in BALB/c mice infected with *L. infantum* mediated by a significant production of IFN-g and suppression of immunoregulatory cytokines, IL-10 and TGF-β, leading to the hypothesis if it is not associated with a decrease in the frequency of regulatory T cells.

The beginning of an appropriate immune response is a challenge to control *Leishmania* infection. Recent approaches to the combination therapy, targeted delivery and use of immunological adjuvants are efforts to reduce the effective dosages of drugs available on the market associated with toxicity (Singh & Sundar 2014). The data from this study open perspectives for the development of new alternative therapeutic formulations for cutaneous leishmaniasis.
